# The Effect of Nucleo-Olivary Stimulation on Climbing Fiber EPSPs in Purkinje Cells

**DOI:** 10.1007/s12311-024-01682-1

**Published:** 2024-03-11

**Authors:** Josefine Öhman, Elias Sjölin, Maurizio Cundari, Fredrik Johansson, Mike Gilbert, Henk-Jan Boele, Pär Svensson, Anders Rasmussen

**Affiliations:** 1 Department of Experimental Medical Science, Lund University, Lund, Sweden; 2 Unit of Neuropsychiatry, Hospital of Helsingborg, Helsingborg, Sweden; 3 Unit of Neurology, Hospital of Helsingborg, Helsingborg, Sweden; 4 School of Psychology, College of Life and Environmental Sciences, University of Birmingham, Birmingham, UK; 5 Princeton Neuroscience Institute, Washington Road, Princeton, USA; 6 Department of Neuroscience, Erasmus MC, 3000 DR Rotterdam, The Netherlands

**Keywords:** Purkinje cells, In-vivo, EPSPs

## Abstract

Climbing fibers, connecting the inferior olive and Purkinje cells, form the nervous system's strongest neural connection. These fibers activate after critical events like motor errors or anticipation of rewards, leading to bursts of excitatory postsynaptic potentials (EPSPs) in Purkinje cells. The number of EPSPs is a crucial variable when the brain is learning a new motor skill. Yet, we do not know what determines the number of EPSPs. Here, we measured the effect of nucleo-olivary stimulation on periorbital elicited climbing fiber responses through in-vivo intracellular Purkinje cell recordings in decerebrated ferrets. The results show that while nucleo-olivary stimulation decreased the probability of a response occurring at all, it did not reduce the number of EPSPs. The results suggest that nucleo-olivary stimulation does not influence the number of EPSPs in climbing fiber bursts.

## Introduction

The brain constantly predicts the future and monitors the accuracy, or error, of these predictions using sensory systems. Learning occurs following errors or when predictions fail [1]. Errors are often graded rather than binary. Rarely do we achieve a perfect outcome on the first attempt, and the necessary change to attain proficiency may be substantial initially but then gradually becomes smaller as we improve. Thus, the brain must have a non-binary code that carries information about error magnitude.

The cerebellum is involved in functions such as timing [2, 3], learning and adaptation of motor programs, and predicting future events – in the motor realm and cognitive tasks. Much of the cerebellum consists of the same recurring neural circuit. Individual microcircuits are computational units whose response to a given context can be adjusted independently [4, 5]. Input to the cerebellum arrives via mossy fibers and climbing fibers. Axons from the pontine nuclei enter the cerebellum as mossy fibers and synapse on granule cells and cells in the cerebellar nuclei. Parallel fibers from granule cells ascend to the molecular layer of the cerebellar cortex, where they synapse on Purkinje cells and inhibitory interneurons. Each Purkinje cell will receive input from an estimated ~ 175.000 granule cells [6, 7]. Climbing fibers are axons from the inferior olive. They ascend to the cerebellar cortex, where they entangle the dendrites of Purkinje cells, with each climbing fiber forming numerous synapses with a Purkinje cell. The connection between the climbing fiber and the Purkinje cell is the strongest in the nervous system [4].

Several studies suggest that climbing fibers transmit information about motor and prediction errors that guide cerebellar plasticity [8–10]. However, the precise cellular mechanisms behind the tuning of motor adaptation have remained elusive and a point of contention [11, 12]. Much research has investigated the role of the climbing fiber signal in eyeblink conditioning. In eyeblink conditioning, a neutral stimulus, transmitted by the mossy fibers, is paired with a reflex-eliciting stimulus sent by the climbing fibers [13–15]. The cerebellum plays a pivotal role in eyeblink conditioning. However, the plasticity responsible for conditioned blink responses remains an active field of research. Eyeblink conditioning induces changes in the cerebellar nuclei [9, 16] and the cerebellar cortex [17–20].

Activation of climbing fibers results in calcium influx throughout the branched dendrites of the Purkinje cell [21], resulting in one to six excitatory postsynaptic potentials (EPSPs). Purkinje cells respond to climbing fiber activation with a complex spike followed by a brief firing pause [22, 23]. The number of EPSPs affects the duration of the pause, although the effect on the complex spike itself is limited [24]. Evidence suggests that the number of spikes in the climbing fiber signal affects the direction of learning in an eyeblink conditioning paradigm [25, 26].

But what determines the number of EPSPs in the Purkinje cell dendrites? Proposed factors include the level of synchronization among olivary cells [27] and the preceding activity in the cerebellar cortex [28]. Moreover, the stimulation strength influences the strength of the calcium signal, which may correlate with the number of EPSPs [29]. Previously, we proposed that activation of the nucleo-olivary pathway [30] also affects the number of EPSPs in the olivary signal [12]. This is in line with evidence that nucleo-olivary stimulation suppresses field potentials elicited by peripheral stimulation [31] and that such stimulation can cause the extinction of a learned response [32, 33]. This might mean nucleo-olivary stimulation can influence the number of EPSPs in climbing fiber responses [34, 35]. However, given the recent discovery that the nucleo-olivary pathway also contains excitatory glutamatergic fibers [36], nucleo-olivary stimulation might also increase the number of EPSPs.

This study aimed to test if nucleo-olivary stimulation affects the number of EPSPs elicited by periorbital stimulation. We hypothesized that periorbital stimulation elicits fewer EPSPs if preceded by nucleo-olivary stimulation.

## Method

### Subjects and surgery

The complete sample consisted of 31 intracellular recordings from cerebellar Purkinje cells in 11 male decerebrated ferrets in-vivo. For some of the analyses, only a subset of these cells were used. The animals were kept under anesthesia until they were decerebrated – rendering them incapable of experiencing pain. After the experiment, the animals were euthanized with an overdose of pentobarbital. This study has been reviewed and approved by the local Swedish Ethical Committee (dnr: 5.8.18–03840/2019).

**Fig. 1:**
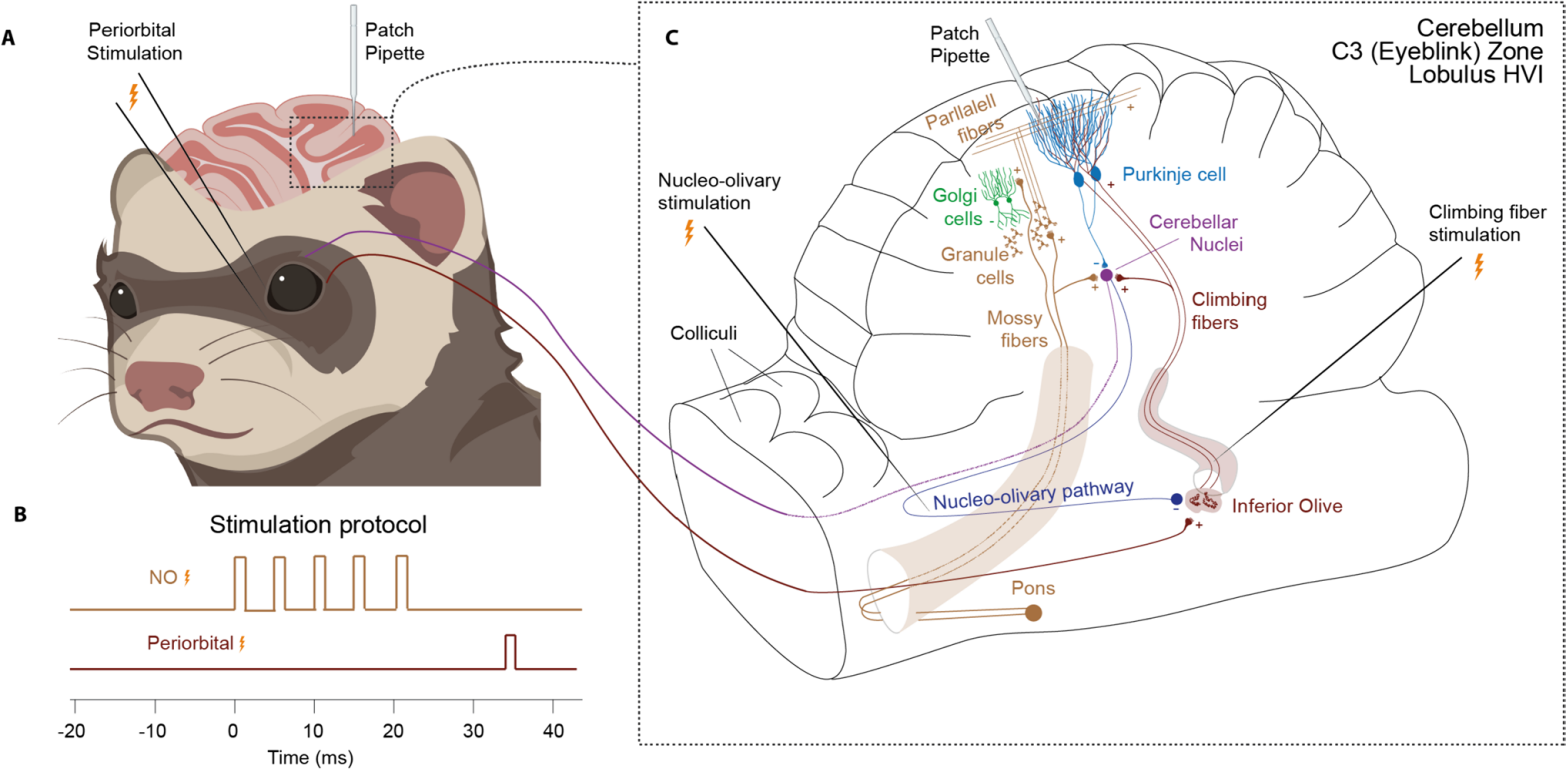
**Experimental setup.** Illustration of the experimental setup including stimulation and recording sites within the cerebellar network. **A.** A sharp glass pipette was inserted into the electrophysiologically identified eyeblink region of the cerebellar cortex. **B.** Stimulation protocol altering between periorbital stimulation and nucleo-olivary + periorbital stimulation. **C.** Enlarged view on the right depicts the cerebellar network including the pathways that different stimuli take within the brain as well as the central stimulation sites

The experimental setup is illustrated in Fig. 1. Ferrets (0.8–1.58 kg) were initially anesthetized with a mixture of O_2_ and air, with 1.5–2% isoflurane (Baxter Medical, Kista, Sweden), subsequently replaced by propofol delivered intravenously. Blood pressure, CO_2_, and temperature were kept within physiological limits throughout the experiment. After the head was fixed in a stereotaxic frame and the skull opened on the left side, the caudal two-thirds of the cerebral hemisphere was removed by aspiration, exposing the anterior cerebellar cortex and the colliculi. Animals were decerebrated by sectioning the brainstem with a blunt spatula 1-2 mm rostral to the superior colliculus. After decerebration, anesthesia was discontinued. With the cerebellum and colliculi exposed, a pool was constructed of cotton-reinforced agar and filled with high-density perfluorocarbon liquid (FC-40 Fluorinert; 3 M, Zwijndrecht, Belgium). To attain the stability needed for intracellular recordings, ferrets were curarized, artificially ventilated, and kept hanging by the spine, with the head fixed in the stereotaxic frame. A bilateral pneumothorax was performed to minimize chest movements.

Since different parts of the cerebellum differ in biochemical properties and functions [4, 37], recordings in this experiment were limited to blink-controlling area of the C3 zone of hemispheral lobule VI, identified by previously established criteria [38, 39]. To locate the blink area on the cerebellar cortex, we recorded EEG activity using a ball electrode (diameter of ~ 2 mm) from the cerebellar cortex while repeatedly applying periorbital stimulation. The blink area was defined based on where periorbital stimulation elicited a field potential with a sharp peak with a delay of 8-12 ms.

### Stimulation

The periorbital region was stimulated using insulated insect needles with a diameter of 1 mm. Periorbital stimulation was kept at a constant intensity of 3 mA, with a duration of 1 ms.

To stimulate the inferior cerebellar peduncle (climbing fibers) and the nucleo-olivary pathway, we used custom-made wolfram stimulation electrodes (diameter, 100 µm; de-insulated tip, 50 µm). To locate and stimulate climbing fibers, the electrode was inserted at a 45° angle, 4 mm lateral to the midline and 4 mm rostral to the caudal border of the cerebellar vermis to a depth of 4.0–5.0 mm. To find a good stimulation site, single 200 µA electrical pulses were delivered at 0.5 Hz while lowering the electrode down through the brain tissue and recording from the cerebellar surface with a 3 mm silver ball. Tracking was stopped once we found a location where the stimulation elicited a distinct field potential with a response latency of 2.0 ms and a low stimulation threshold (< 30 µA). If a specific track did not yield any clear climbing fiber field potential, we moved the electrode 1 mm laterally and 1 mm rostrally.

The nucleo-olivary pathway was stimulated by lowering a stimulation electrode into the superior cerebellar peduncle 1 mm from the midline at the caudal border of the inferior colliculus. The ability to depress periorbital evoked climbing fiber field potentials was tested by repeatedly applying five pulses (0.1 ms; 200 Hz, 100 μA), followed by one periorbital pulse (1 ms; 2–3 mA) 35 ms later. The optimal stimulation site was found by comparing the field potential elicited by the periorbital stimulus with and without the preceding superior cerebellar peduncle stimulation. Tracking was stopped when stimulation resulted in significant suppression of the periorbital field potential (> 50%) with a low stimulation threshold (< 100 μA). In the testing phase, the nucleo-olivary pathway was stimulated with 5 pulses of 0.1 ms duration at an intensity between 10–100 µA.

### Cell recordings

Intracellular recordings were made in-vivo in the superficial layer of the eyeblink region of the cerebellar cortex. Sharp pipettes were pulled from borosilicate glass capillaries to 10–20 MOhm using a Sutter Instruments (Novato, CA) P-30 vertical puller. The pipettes were filled with an electrolyte solution. The pipette was slowly inserted into the tissue at approximately 3 µm per second. When an extracellular Purkinje cell recording was obtained (based on the presence of simple and complex spikes), we verified that the cell responded to periorbital and climbing fiber stimulation before attempting to penetrate the cell. To penetrate the membrane, we first slowly advanced the pipette so that it was on or very close to the membrane. Then we tried to penetrate the Purkinje cell using a subtle jabbing movement of the pipette.

### Data analysis

**Fig. 2:**
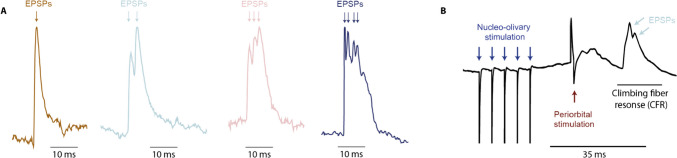
**Raw data traces. A.** Examples of climbing fiber responses with variable numbers of EPSPs. **B**. Stimulation example in which nucleo-olivary stimulation is followed by periorbital stimulation

Data was sampled and later analyzed using Spike2 version 9. EPSPs following stimulation were identified and sorted by stimulation type, strength, and number of EPSPs for each cell (Fig. 2). Some EPSPs were excluded based on their undefined form, usually due to recording circumstances, making their accurate classification impossible. At first, templates and wavemarks were used for the classification of EPSPs. However, the method was unreliable compared to manual quantifying due to the imprecise classification of EPSPs. To increase the accuracy of EPSP quantification, two independent individuals compared their results on the number of EPSPs after each stimulation. Only cells that were judged with a high degree of similarity were kept for the subsequent analysis.

When analyzing biological data, the issue of dependency within clusters of data points is not uncommon. In our dataset, the variation between many cells was larger than the variation within each cell. To incorporate this variance between cells when examining the effect that nucleo-olivary stimulation had on the number of EPSPs, a linear mixed effects model was applied. Unlike a traditional linear model that only allows for fixed effects, a mixed-effects model allows for the incorporation of random effects, such as differences in means between groups. If not accounted for, such factors may increase the risk of type 1 and type 2 errors. The model uses all available data points to estimate the effect of chosen values as well as a p-value [40]. Here, we used nucleo-olivary stimulation intensity as a fixed effect and the cell as a random effect.

## Results

### Descriptive statistics

The final dataset consisted of intracellular recordings from 31 Purkinje cell dendrites in 11 decerebrated ferrets. These recordings yielded 1175 observations with 0–6 EPSPs – including spontaneous climbing fiber responses, stimulation-elicited climbing fiber responses, and lack of responses following stimulation (0 EPSPs). The number of stimulations per cell varied from 5–81, mostly due to the varying length of cell recordings. The variable length of the recordings presented a challenge when determining the data distribution. In some cells, the distribution of EPSPs was normally distributed, but when combing all data points, the data visually resembled a negative binomial distribution. To decide whether to treat the data as normally distributed, the distribution of each cell was first inspected visually, and then we created a Cullen and Frey graph to compare the skewness and kurtosis of each cell. Based on these procedures, we treated the data as normally distributed, which also gave the best model fit compared to others using Q-Q plots. Even though the periorbital stimulation strength was constant, the number of EPSPs in the resulting climbing fiber responses varied significantly (p < 0.001) between cells even without nucleo-olivary stimulation. Due to the variation between cells, we decided to use a linear mixed-effects model. Using linear correlation or ANOVA would require averages across different cells, whereas a mixed effects model allows us to measure changes within each cell without resorting to averages.

Due to the variable duration of the recordings and the differing placement of the pipette relative to the cell soma, the data present in each recording varied. To compare EPSPs in spontaneous and stimulation-elicited climbing fiber responses, cells needed to exhibit both types of responses. To evaluate the impact of nucleo-olivary stimulation on climbing fiber responses, the recordings needed to be long enough to include instances of both periorbital stimulation alone and periorbital stimulation preceded by nucleo-olivary stimulation. As a result, different analyses were conducted on various subsets of the total sample, which consisted of 31 intracellular recordings.

### Cells have different numbers of EPSPs in spontaneous climbing fiber responses

**Fig. 3:**
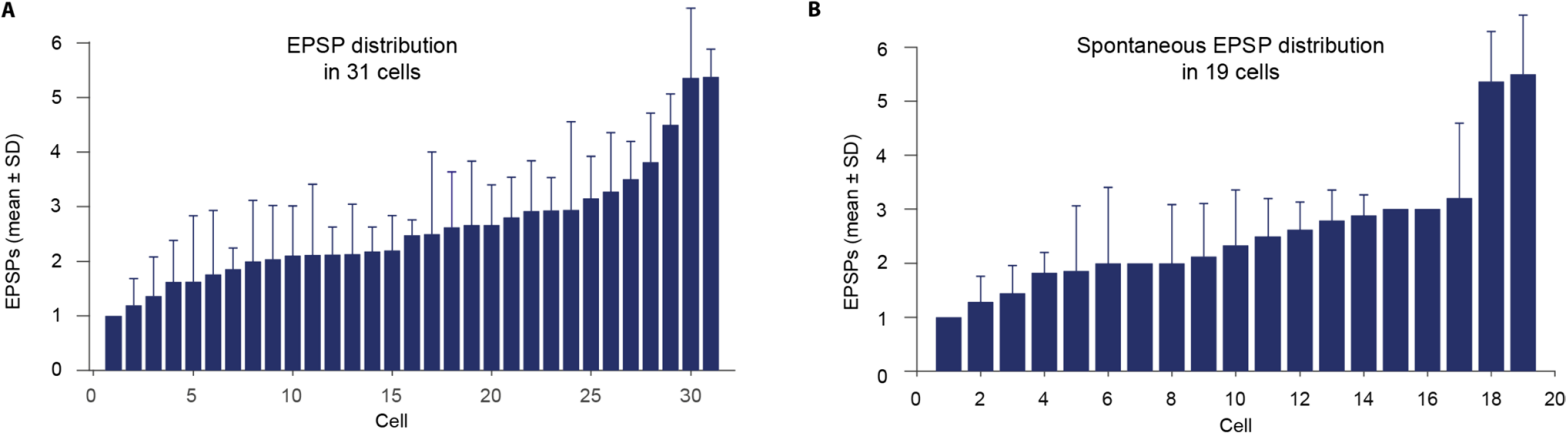
**Different cells have different EPSP distributions. A.** Mean ± SD of EPSPs in individual cells. **B.** Distribution of EPSPs in cells with spontaneous climbing fiber responses

**Fig. 4:**
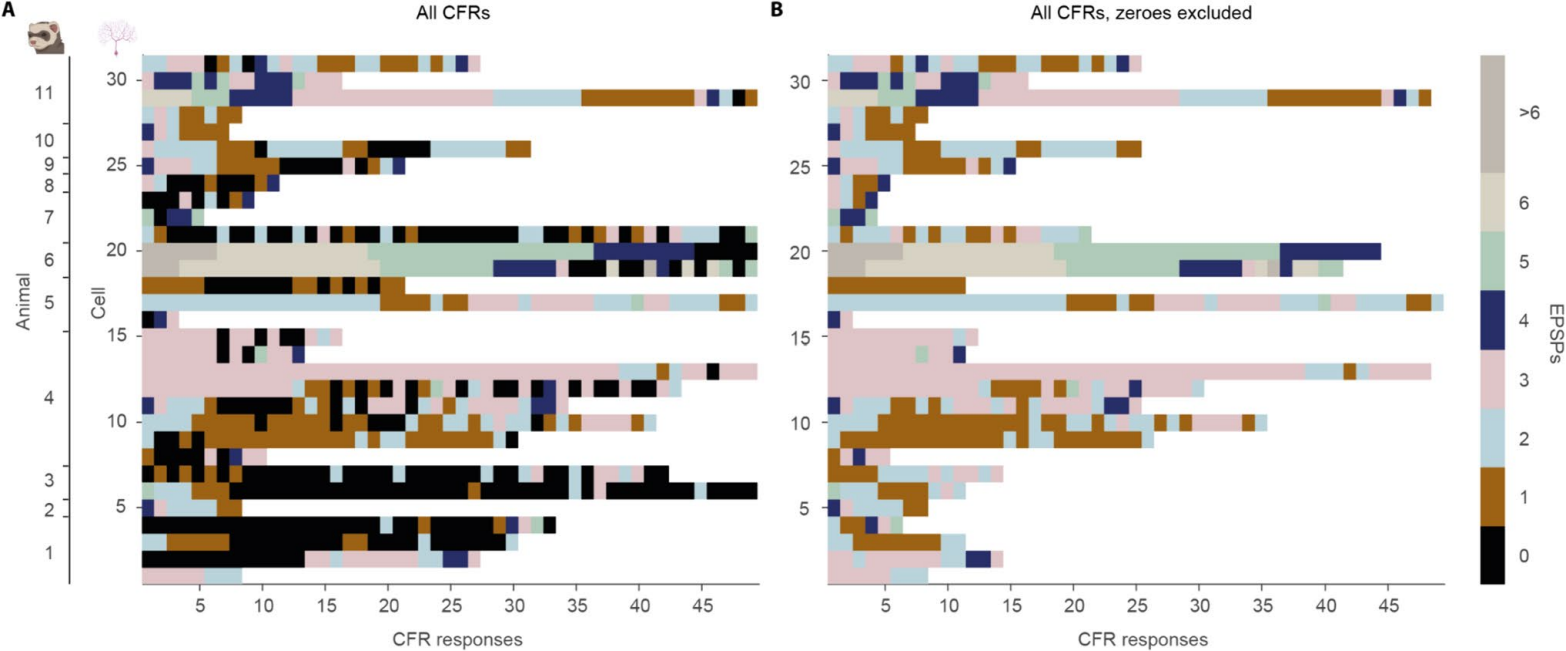
**Number of EPSPs in climbing fiber responses.** Each row shows a different cell and columns are separate climbing fiber responses, or absence of climbing fiber responses following stimulation. The color of the pixels indicates the number of EPSPs in the climbing fiber response. **A.** Data including instances where stimulation did not elicit a response, for example when NO stimulation suppressed the climbing fiber response or where periorbital stimulation did not elicit a response. **B.** The same data but with all zeroes removed

To determine if cells varied in terms of the average number of EPSPs in spontaneous climbing fiber responses, we performed a one-way ANOVA comparing the EPSP distribution of the 19 cells from which we had clear spontaneous climbing fiber responses. The one-way ANOVA showed that the distribution of spontaneous EPSPs varied significantly between cells [F(18) = 37.63, p < 0.0001, eta-squared (η^2^) = 0.723]. The pattern that different cells differ in their typical number of EPSPs is also evident in Fig. 3 and Fig. 4.

### No difference in spontaneous and elicited climbing fiber responses

To test if there was a difference in the number of EPSPs between spontaneous and stimulus-elicited climbing fiber responses, we analyzed the subset of cells with clear instances of both spontaneous and elicited climbing fiber responses (n = 15). A paired t-test revealed no statistically significant difference in the number of EPSPs between spontaneous and elicited climbing fiber responses [t(14) = 0.219, p = 0.83, Cohen’s d = 0.023].

### Nucleo-olivary stimulation probability of climbing fiber responses but not the number of EPSPs

**Fig. 5:**
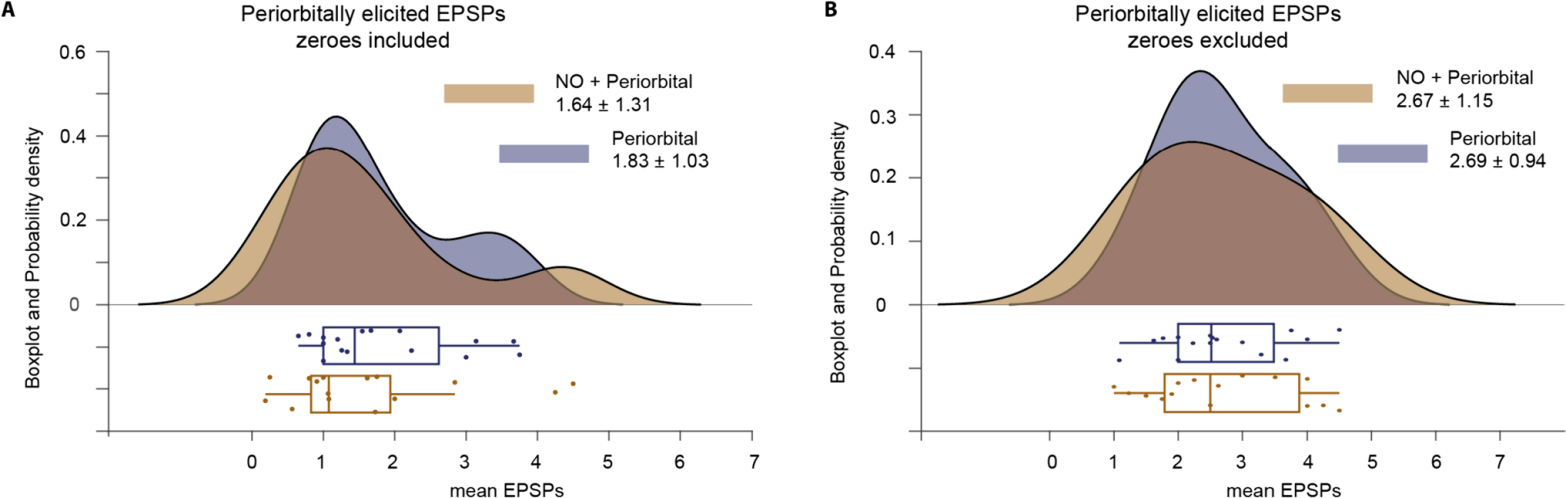
**Effect of nucleo-olivary stimulation on CFR EPSP distribution. A.** Comparison of EPSP counts in peri-orbitally elicited climbing fiber responses with or without preceding nucleo-olivary stimulation. **B.** Same as A, but with all trials in which the periorbital stimulation did not elicit a response were excluded. Both figures display probability density function (PDF), and boxplots using raincloud plots package for Matlab (see [41] for details)

To test whether nucleo-olivary stimulation affected climbing fiber responses in Purkinje cells, we used a linear mixed-effects model with nucleo-olivary stimulation strength as a fixed effect and cell id as a random effect. We performed this analysis on a subset of cells (n = 16), where recordings were sufficiently long to compare climbing fiber responses with and without nucleo-olivary stimulation. The linear mixed-effects model revealed that stimulation strength suppressed the climbing fiber response by 0.005 EPSPs per µA (p = 0.004). At first glance, this would seem to support our hypothesis that nucleo-olivary stimulation reduces the number of EPSPs. However, another possibility is that this effect reflected a complete suppression of the olivary response, that is, 0 EPSPs. To test this, we used a second linear mixed-effects where we excluded all trials in which the periorbital stimulation did not elicit any response. In this second model, stimulation strength had a smaller effect – only 0.001 EPSPs per µA, which was not statistically significant (p = 0.524). Together, these results suggest that electrical stimulation of the nucleo-olivary pathway reduces the probability of a climbing fiber response but not the number of EPSPs within that response (see Fig. 5).

## Discussion

The aim of this study was to test if activation of the nucleo-olivary pathway reduces the number of EPSPs in climbing fiber responses in cerebellar Purkinje cells. We did not find support for this hypothesis. While electrical stimulation of the nucleo-olivary pathway did reduce the probability that stimuli trigger a climbing fiber response, nucleo-olivary stimulation did not alter the number of EPSPs in the climbing fiber responses.

Our results show that the number of EPSPs in climbing fiber responses vary between cells. This is consistent with earlier studies performed on several species, including rabbits [42], rats [27], and ferrets [26]. The results show that spontaneous climbing fiber responses and climbing fiber responses, induced by periorbital stimulation, elicit a variable number of EPSPs in the receiving Purkinje cells. The exact number of EPSPs varies between 1–7 EPSPs, with some cells consistently having more EPSPs than others. The considerable variation between cells suggests that the number of EPSPs is not merely a result of the subthreshold olivary oscillations, as has been previously proposed [27]. Had that been the case, we should expect all or most cells to have climbing fiber responses with a greater variation of EPSPs. Additionally, factors such as the exact placement of electrodes in different experiments could contribute to the variation in the average number of EPSPs per stimulation. Individual cells may also express natural differences in their excitability when responding to periorbital stimulation [43].

Several studies suggest that stimulation-induced climbing fiber activation yields more EPSPs [42], and a larger calcium influx [29] than spontaneous climbing fiber activation. Contrary to our prediction we did not find a significant difference in the number of EPSPs between stimulation elicited and spontaneous climbing fiber responses. More studies will be necessary to elucidate the relationship between calcium influx and the number of EPSPs. It might still be the case that a larger calcium influx [29] does not lead to more EPSPs. It is difficult to tell to what degree subtle experimental conditions, such as the exact placement of the electrode, influence the results. It is also difficult to evade the conclusion that the variation between cells, at least partly, reflects the intrinsic properties of the cells themselves. This variability, combined with the fact that all our cells were in the C3 microzone, suggests that Purkinje cells in the same functional microzone vary significantly in how they respond to climbing fiber input. This is consistent with the recent observation that simple spike synchrony between Purkinje cells in the same microzone is low [44].

Our two linear mixed effects models revealed that electrical stimulation of the nucleo-olivary pathway reduces the probability that subsequent peripheral stimuli induce a climbing fiber response. Contrary to our prediction, it does not appear to affect the number of EPSPs in the Purkinje cell. If nucleo-olivary stimulation does not alter the number of EPSPs in the climbing fiber response, the next question becomes what determines the number of EPSPs. Perhaps the phase of the olivary sub-threshold oscillations determines the number of EPSPs [27]? Oscillations may, in turn, be influenced by cerebellar mechanisms such as the nucleo-olivary pathway, which has been shown to influence the gap junctions between olivary neurons [45]. The absence of EPSP modulation could also mean that electrical stimulation is not sufficiently nuanced to nudge nucleo-olivary inhibition. One possible interpretation of our data is that, unless perfectly tuned, electrical stimulation will not affect climbing fiber responses. An alternative is that learned Purkinje cell pause responses could affect the number of EPSPs via disinhibition of the cerebellar nuclei. Purkinje cells converge at the level of cerebellar nuclei [46]. A single cell may have a small effect. Grading error signals could, in theory, be achieved by controlling the number of climbing fibers activated. This might, in turn, modify the response of microzone-grouped Purkinje cells as a population [47].

Though not quantitatively analyzed, the distribution of EPSPs (Fig. 4D) hints that nucleo-olivary stimulation results in more extreme values. One possible explanation is that – due to the short distance between the stimulation site and the climbing fibers – increasing the stimulation strength leads to an increased risk of spillover olivary excitation, which could counter inhibitory effects. Alternatively, the recent finding that the nucleo-olivary pathway contains both inhibitory GABAergic and excitatory glutamatergic fibers [36] could also explain why stimulation would result in more extreme values. However, the most straightforward interpretation is that activation of the nucleo-olivary pathway does not influence the number of EPSPs in climbing fiber responses.

Motor learning typically entails a long series of trials with gradually diminishing errors. We do not know how error magnitude is encoded in the cerebellum, but one possibility is that the number of EPSPs in climbing fiber responses encodes this information and that nucleo-olivary activation can modulate the number of EPSPs as learning progresses. The results from this experiment suggests that this is not the case because electrical activation of the nucleo-olivary pathway did not alter the number of EPSPs in the climbing fiber responses. Whether this is because the nucleo-olivary pathway cannot alter the number of EPSPs or because electrical stimulation is too blunt an instrument to answer this question must be addressed in future research.

## Data Availability

The data and code used for this paper is available at https://github.com/rasmussenanders/CFR.
